# Evaluation of the combined use of two different respiratory monitoring systems for 4D CT simulation and gated treatment

**DOI:** 10.1002/acm2.12434

**Published:** 2018-08-13

**Authors:** Jie Liu, Teh Lin, Jiajin Fan, Lili Chen, Robert Price, C‐M Charlie Ma

**Affiliations:** ^1^ Department of Radiation Oncology Fox Chase Cancer Center Philadelphia PA USA

**Keywords:** 4D CT, ANZAI belt, gating, respiratory monitoring systems, RPM

## Abstract

**Purpose:**

Two different respiratory monitoring systems (Varian's Real‐Time Position Management (RPM) System and Siemens’ ANZAI belt Respiratory Gating System) are compared in the context of respiratory signals and 4D CT images that are accordingly reconstructed. This study aims to evaluate the feasibility of combined use of RPM and ANZAI systems for 4DCT simulation and gated radiotherapy treatment, respectively.

**Methods:**

The RPM infrared reflecting marker and the ANZAI belt pressure sensor were both placed on the patient's abdomen during 4DCT scans. The respiratory signal collected by the two systems was synchronized. Fifteen patients were enrolled for respiratory signal collection and analysis. The discrepancies between the RPM and ANZAI traces can be characterized by phase shift and shape distortion. To reveal the impact of the changes in respiratory signals on 4D images, two sets of 4D images based on the same patient's raw data were reconstructed using the RPM and ANZAI data for phase sorting, respectively. The volume of whole lung and the position of diaphragm apex were measured and compared for each respiratory phase.

**Results:**

The mean phase shift was measured as 0.2 ± 0.1 s averaged over 15 patients. The shape of the breathing trace was found to be in disagreement. For all the patients, the ANZAI trace had a steeper falloff in exhalation than RPM. The inhalation curve, however, was matched for nine patients, steeper in ANZAI for five patients and steeper in RPM for one patient. For 4D image comparison, the difference in whole‐lung volume was about −4% to +4% and the difference in diaphragm position was about −5 mm to +4 mm, compared in each individual phase and averaged over seven patients.

**Conclusions:**

Combined use of one system for 4D CT simulation and the other for gated treatment should be avoided as the resultant gating window would not fully match with each other due to the remarkable discrepancy in breathing traces acquired by the two different surrogate systems.

## 1 INTRODUCTION

Management of respiratory motion is an important component in the workflow of thoracic and abdominal radiotherapy.^1^, ^2^, ^3^ 4D CT incorporates the patient's respiratory information into a stack of 3D images such that the sequential snapshot images at different respiratory phases can be reconstructed.^4^, ^5^ With the 4D images, the tumor excursion range with respiration can be obtained and a patient‐specific internal margin can be decided for contouring the internal tumor volume.^1^, ^6^, ^7^ Respiratory gating in radiation delivery provides effective motion control by disabling radiation beam when respiration magnitude exceeds certain threshold, which is beneficial for reducing the planned target size and sparing more healthy tissues.^8^, ^9^, ^10^

4D CT acquisition requires oversampling CT data for the same slices such that images at different respiratory phases can be reconstructed over a full breathing cycle, which is known as the data sufficiency condition.^11^ The 3D volumetric images at individual respiratory phases are obtained by sorting the oversampled images or sinograms based on the associated respiratory phase‐angle or amplitude from the breathing trace that is acquired simultaneously during scanning.^12^ In the treatment planning process, if a gated treatment plan is decided, the 4D images are combined with the respiratory motion data to select an appropriate gating window.^10^ Several respiratory monitoring systems using external surrogates are available in the market for the purposes of measuring respiratory motion for 4D CT imaging and gated treatment.^5^ These systems measure the respiratory motion as the surface movement of abdomen or chest wall (eg, Real‐Time Position Management [RPM],^4^ C‐RAD,^13^ GateCT^14^), or the pressure change in a belt wrapped around the abdomen (eg, ANZAI,^15^ Bellows^16^), etc. There is no consensus of which is the best external surrogate with respect to the correlation with the internal tumor motion.

In our institution, both RPM (Varian Medical Systems, Palo Alto, CA, USA) and ANZAI belt systems (Anzai Medical Co. Ltd, Tokyo, Japan) are used for 4D CT image acquisition while only RPM is available for respiratory gated treatment. In the scenario of mixed use of the ANZAI system for 4D CT and RPM for treatment gating, the differences between the two systems may be detrimental to the desired clinic outcome. Previous studies^14^, ^15^, ^16^ compare different types of surrogates based on phantom experiments and show high agreement achievable for both regular and irregular breathing patterns. However, human respiratory motion involves movement in three dimensions rather than the 1D movement as designed in the phantom studies. De Groote^17^ proposed a model showing that if there exists asynchrony between motions along the dorsoventral and lateral directions with respiration, there will be a phase shift between the anterior–posterior movement (as measured as the RPM signal) and the change in abdominal perimeter (as measured as the ANZAI belt signal). In addition, in previous studies comparing different external surrogates,^13^, ^16^, ^18^ shape distortion can be observed between the respiratory traces in patients, which has not been investigated in those studies.

The purpose of this study is to evaluate the discrepancies between the ANZAI and RPM systems by comparing the respiratory signals and the corresponding 4D images reconstructed separately. The feasibility of combined use of the two surrogates for 4D CT and treatment gating is also discussed.

## 2 MATERIALS AND METHODS

### 2.A Simultaneous acquisition of respiratory signal by RPM and ANZAI

**Figure 1:**
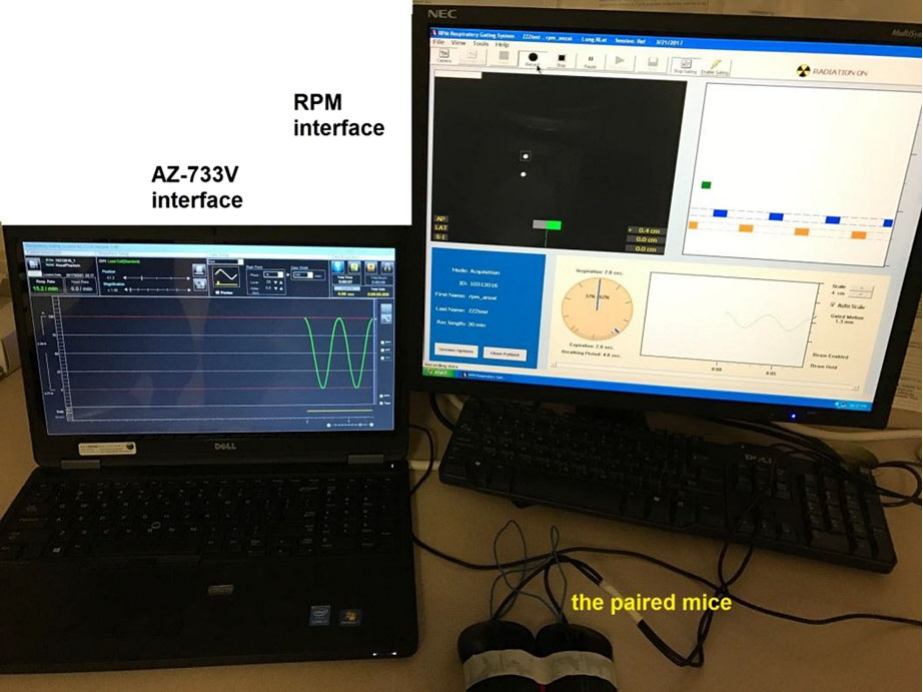
The paired mice control the start of data collection on ANZAI and RPM interfaces simultaneously by a single left click.

Fifteen patients with lung cancer were enrolled for respiratory signal comparison with 4D CT scan on a Siemens SOMATOM Definition AS (Open 20 RT) (helical scan mode, source rotation time 0.5 s or 1 s). The CT scanner can receive respiratory signal from either the RPM system or the ANZAI system for sorting sinogram to reconstruct 4D images. When the ANZAI system is connected to the scanner as the online respiratory monitoring system, the ANZAI data are integrated into the CT raw data, making it difficult to retrieve the ANZAI respiratory signal for offline analysis. Instead, when the RPM system is used, the RPM data file is saved separately and can be easily imported into the CT workstation through an open interface. In this study, both the RPM and ANZAI systems were placed on the same patient during 4D CT scan, with the RPM connected to the scanner as the online respiratory monitoring system while the ANZAI signal was collected independently by a laptop using the AZ‐733V software (ANZAI Medical). The RPM infrared reflecting marker box and the ANZAI pressure sensor were placed between the umbilicus and xiphoid process and adjacent to each other to avoid the potential impact of surrogate positioning difference on the respiratory signal. Data collection was started by clicking the “Record” icon on the RPM interface and the “Start” icon on the AZ‐733V interface. To synchronize the respiratory signal acquired by the two systems, the left button of the two mice that were each connected to each computer were soldered together such that a single click on either mouse can start data collection on both systems simultaneously. Figure 1 shows the interfaces of the RPM workstation and the AZ‐733V software, as well as the connection of the paired mice.

### 2.B Analysis of respiratory data

After completion of 4D CT scans, the respiratory data files were exported for analysis in MATLAB (MathWorks Inc., Natick, MA, USA). The peaks (end‐of‐inhalation [EOI]) and valleys (end‐of‐exhalation [EOE]) of the breathing traces were determined by searching the local maxima and minima with a moving searching range less than half breathing period. Further manual adjustment for the positions of EOI/EOE was enabled by programming the maxima/minima as draggable points on the traces. The breathing period was calculated as the time difference between adjacent EOI points and the breathing depth was calculated as the amplitude increment from EOE to EOI. The linear correlation coefficient was calculated between the ANZAI and RPM data over the entire traces.

The phase shifts between the two traces were calculated as the time latency to reach the EOI. To compare the trace shape between ANZAI and RPM, each individual breathing cycle between two adjacent EOE were extracted and plotted together. The amplitude was renormalized from 0 to 100 for each cycle and all cycle curves were aligned at the EOI. The average trace shape within one breathing cycle was obtained by averaging the amplitude of the aligned curves as a function of time.

### 2.C Comparisons on 4D images

**Figure 2:**
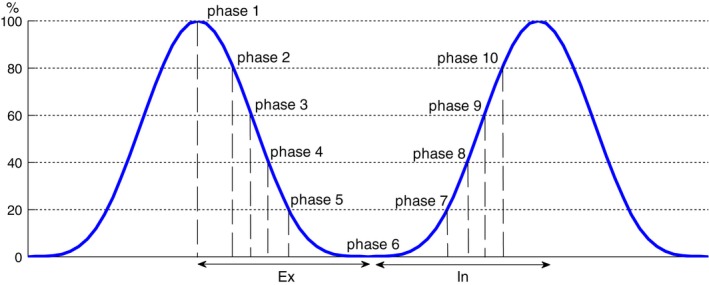
Illustration of local amplitude based phase sorting. Phases 1–10 correspond to 100%Ex to 80%In.

As the open interface mode is used for receiving respiratory signal from RPM, the CT workstation only allows respiratory files in RPM data format to be imported. For comparisons of 4D images reconstructed using both surrogates, the ANZAI file was converted into the RPM file format with the relative pressure measurement replacing the anterior–posterior movement as in the RPM data lines. Besides, the ANZAI file did not contain the real‐time records of CT beam on/off indicators as it was not connected to the scanner during scanning. The TTLin (CT on) and TTLout (CT off) indicators in the ANZAI data lines were obtained from the corresponding RPM data lines with the timestamps aligned. The RPM file and the converted ANZAI file were sequentially imported into the CT workstation to reconstruct two separate sets of 4D images with the same CT raw data (sinogram). The local amplitude sorting algorithm was used for acquiring images at 10 respiratory phases (Fig. 2). The two sets of 4D images were exported to the Eclipse (Varian Medical Systems, Palo Alto, CA, USA) treatment planning system for comparisons. Seven of the 15 patients were selected to reconstruct two sets of 4D images using the RPM and ANZAI respiratory files, respectively. Image fusion was performed phase by phase for visual observation of differences in anatomy and tumor contour. The whole lung was segmented in all 10 phases on the two sets of 4D images. The volumes of the whole lung were calculated. As the largest respiratory motion is generally seen at the apex of diaphragm, its axial positions in different respiratory phases were used as indicating the correlation between the internal organ motion and external surrogates. The axial positions of diaphragm apex in each set of 4D images were measured and the differences were calculated.

## 3 RESULTS

### 3.A Correlation between the respiratory signal acquired by ANZAI and RPM systems

**Figure 3:**
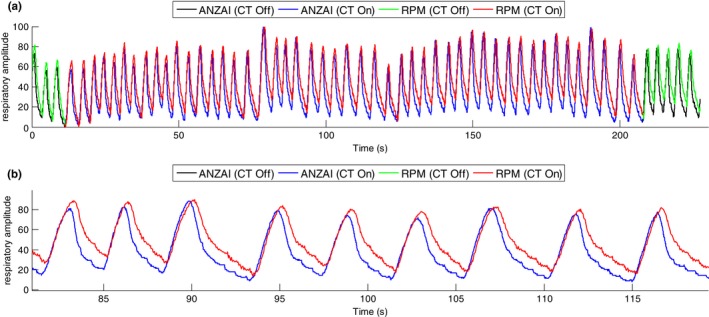
(a) The RPM and ANZAI traces of patient #1, simultaneously acquired during 4D CT scan. The CT beam on/off is marked with different colors (Off: Black (ANZAI) and Green (RPM), On: Blue (ANZAI), Red (RPM)). (b) A portion of the traces between 80 and 120 s, showing the misalignment in details.

**Figure 4:**
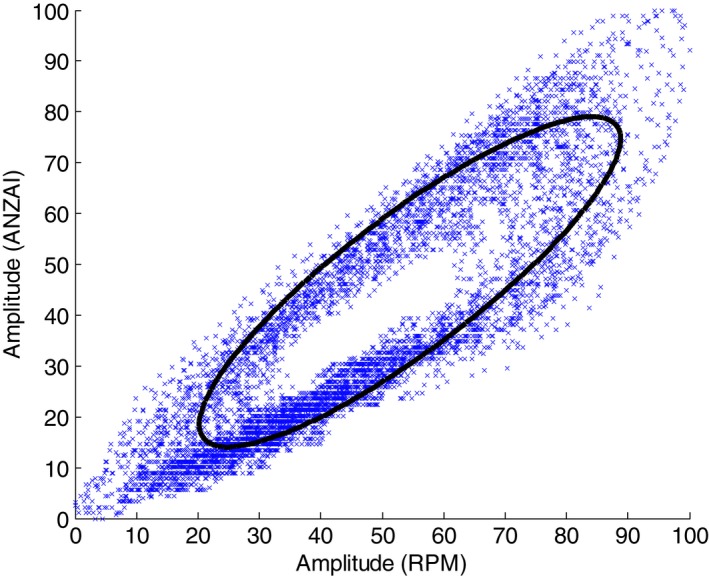
Correlation scatter plot for the RPM and ANZAI traces that are shown in Figure 3(a). An elliptical fitting for the scatter plot is also shown, indicating the existence of a phase shift between the ANZAI and RPM traces (see discussions in Section 4).

The synchronized ANZAI and RPM data were plotted together with the signal amplitude renormalized from 0 to 100 in the range of the entire traces. Figure 3(a) shows the respiratory traces from patient #1, with CT beam on/off marked on both traces (scan time 190 s, trace length 230 s). A portion between 80 and 120 s is enlarged in Fig. 3(b) and shows the apparent misalignment between the two traces. Figure 4 shows the correlation scatter plot with each point consisting of the respiratory amplitude of the ANZAI (the vertical axis) and RPM (the horizontal axis) data at the same sampling time. The linear correlation coefficient was calculated to be 0.87.

For all the 15 patients, the respiratory period (T) and breathing depth (d) measured by ANZAI and RPM are listed on Table 1. The measurements for T are in high agreement while relatively large discrepancies are seen on d measurement. The renormalization method using the global maximum and minimum in the entire trace may enlarge the difference in breathing depth. Table 1 also lists the linear correlation coefficients between the ANZAI and RPM data for each patient.

**Table 1:** The respiratory period (T) and breathing depth (d) measured by ANZAI and RPM for fifteen patients are summarized. The ranges of the differences in period (T_ANZAI_−T_RPM_) and depth (d_ANZAI_−d_RPM_) compared in each individual respiratory cycle are also shown. The linear correlation coefficients (*r*) between ANZAI and RPM are shown in the last column

Patient	T_ANZAI_ (s)	T_RPM_ (s)	T_ANZAI_−T_RPM_ (s)	d_ANZAI_ (%)	d_RPM_ (%)	d_ANZAI_−d_RPM_	*r*
	avg ± std	avg ± std		avg ± std	avg ± std		
1	4.06 ± 0.54	4.06 ± 0.53	(−0.11, +0.06)	65 ± 9	60 ± 7	(−8, +14)	0.87
2	3.27 ± 0.24	3.27 ± 0.23	(−0.06, +0.04)	71 ± 11	58 ± 10	(+5, +19)	0.72
3	5.50 ± 0.92	5.50 ± 0.92	(−0.09, +0.06)	82 ± 10	70 ± 9	(+7, +17)	0.89
4	3.21 ± 0.35	3.22 ± 0.35	(−0.04, +0.04)	57 ± 11	60 ± 11	(−10, +9)	0.83
5	4.15 ± 0.58	4.15 ± 0.58	(−0.09, +0.08)	61 ± 7	64 ± 6	(−12, +22)	0.93
6	5.61 ± 1.77	5.61 ± 1.77	(−0.10, +0.09)	68 ± 14	67 ± 13	(−3, + 9)	0.94
7	4.06 ± 0.59	4.07 ± 0.59	(−0.08, +0.05)	53 ± 13	67 ± 15	(−24, −5)	0.94
8	2.81 ± 0.64	2.82 ± 0.65	(−0.17, +0.16)	67 ± 18	36 ± 15	(−1, +41)	0.87
9	5.28 ± 1.58	5.27 ± 1.60	(−0.07, +0.04)	66 ± 17	59 ± 16	(−1, +15)	0.90
10	3.92 ± 0.50	3.92 ± 0.50	(−0.08, +0.08)	58 ± 7	54 ± 8	(−4, +12)	0.79
11	2.55 ± 0.11	2.55 ± 0.12	(−0.06, +0.07)	66 ± 8	75 ± 7	(−21, +5)	0.75
12	2.70 ± 0.55	2.71 ± 0.55	(−0.12, +0.10)	60 ± 15	47 ± 16	(−3, +20)	0.93
13	4.36 ± 0.68	4.37 ± 0.70	(−0.07, +0.07)	73 ± 13	68 ± 13	(−4, +15)	0.95
14	3.06 ± 0.34	3.06 ± 0.32	(−0.21, +0.18)	65 ± 10	57 ± 9	(−7, +21)	0.66
15	3.02 ± 0.27	3.02 ± 0.27	(−0.30, +0.29)	68 ± 8	72 ± 7	(−9, +1)	0.91

### 3.B Phase shift between ANZAI and RPM traces

**Figure 5:**
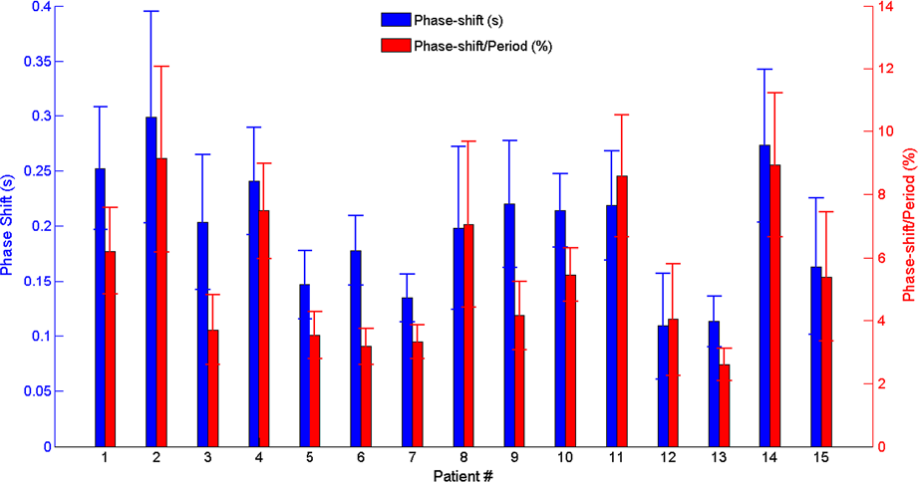
The phase shifts between ANZAI and RPM traces measured in second (blue bar) and percentage of respiratory period (red bar) for 15 patients. The bar and errorbar represent the mean and standard deviation values, respectively.

As shown in Fig. 3, the ANZAI trace leads the RPM trace in time, introducing a phase shift between the two traces. The phase shift was measured as the time difference to reach the EOI within each breathing cycle in RPM compared to ANZAI. The phase shift (mean value and standard deviation) for each patient is plotted in Fig. 5 with positive values representing that ANZAI reaches EOI ahead of RPM in time. The phase shift averaged over all the 15 patients is 0.2 ± 0.1 s. The phase shift calculated as percentage of respiratory period is also plotted in Fig. 5, with the mean value 5.5% ± 2.3% averaged over 15 patients.

### 3.C Distortions in respiratory trace shape

**Figure 6:**
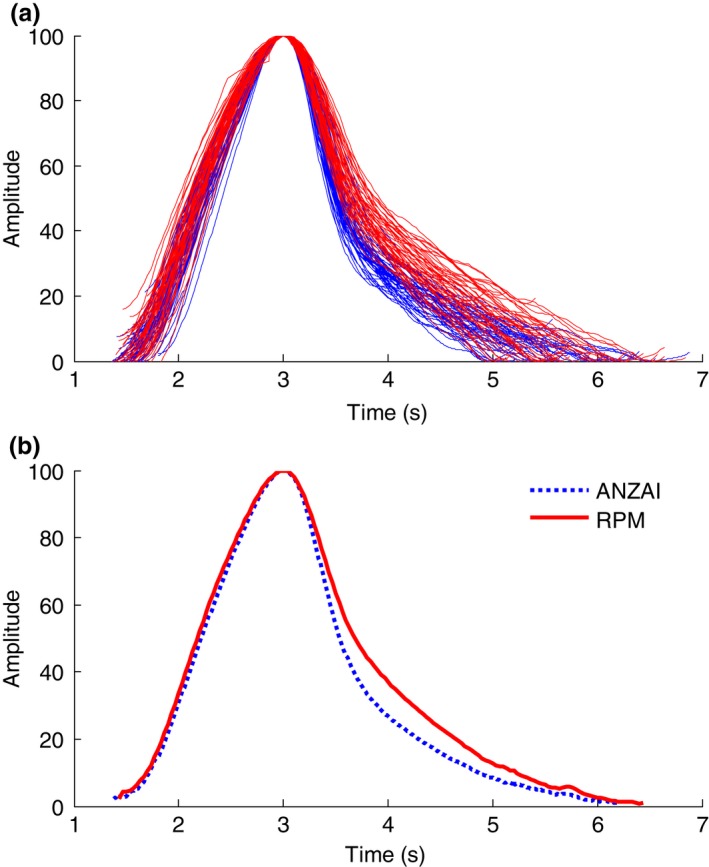
(a) Overlapped plots of individual breathing cycles (the original respiratory traces are shown in Figure 3 for patient 1) (blue lines: ANZAI, red lines: RPM). (b) The average shape of breathing cycle (dashed blue: ANZAI, solid red: RPM).

Another major discrepancy between ANZAI and RPM is the distortion in the trace shape. Figure 6(a) shows the overlapped plots of all individual breathing cycles of ANZAI and RPM, extracted from the respiratory traces as shown in Fig. 3(a). Each breathing cycle is defined as the trace in between two sequential end‐of‐exhalation points and its amplitude is renormalized to the local maximum which is at the end‐of‐inhalation. All the breathing cycles are aligned at the EOI. The average breathing cycle shape of ANZAI and RPM are shown in Fig. 6(b). The inhalation curves are in high agreement, whereas the exhalation curve of ANZAI is steeper than that of RPM.

**Figure 7:**
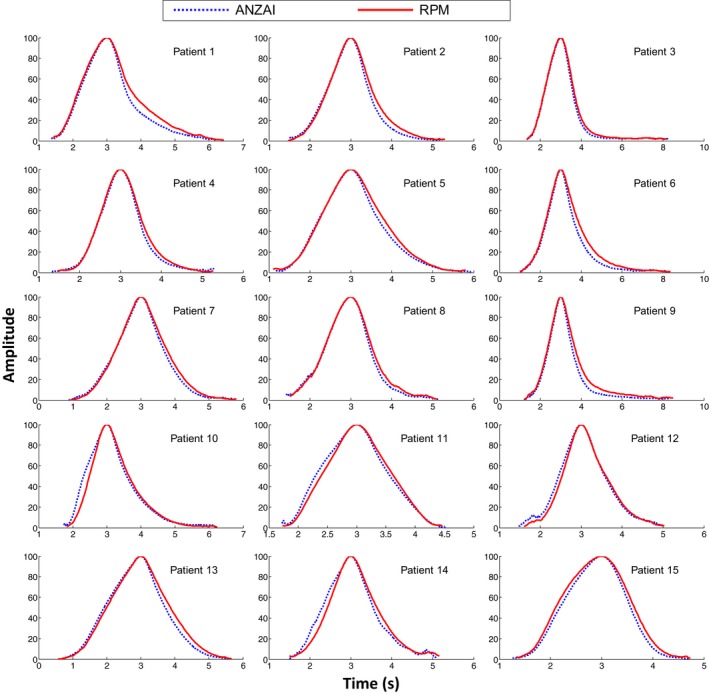
The average breathing cycle shape of the respiratory traces acquired by the ANZAI (dashed blue) and RPM (solid red) for 15 patients.

For all 15 patients, the average breathing cycle shapes are shown in Fig. 7. Patients #2–9 are similar to patient #1 with a steeper falloff in exhalation measured by ANZAI than RPM. For patients #10–14, the inhalation curve of ANZAI is also steeper than that of RPM. For patient #15, in contrast, the inhalation curve of ANZAI is relatively more gradual than that of RPM. But the exhalation curve is still steeper in ANZAI than RPM, consistent with all the other patients.

### 3.D Comparisons of 4D images

**Figure 8:**
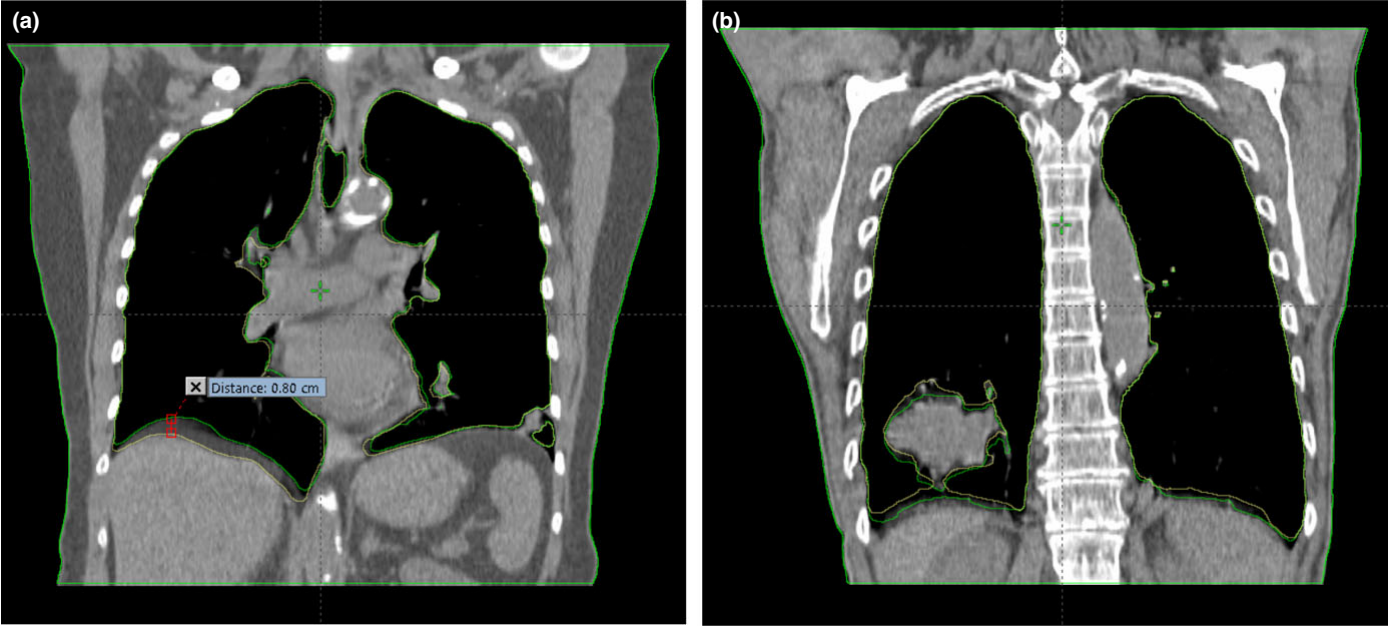
Fused images that are reconstructed using respiratory traces by ANZAI (yellow contour) and RPM (green contour): (a) phase 4 of patient #1, (b) phase 7 of patient #6.

Image comparisons were performed for seven patients (patient #1, #2, #3, #10, #11, #12 and #15), each with two sets of 4D images reconstructed using the ANZAI and RPM data, respectively. The seven patients were selected from the three groups as described in above (Section 3.C.) with respect to the difference in inhalation curve. The local amplitude sorting algorithm was applied to obtain the images for 10 respiratory phases. The scheme of local amplitude sorting is illustrated on Fig. 2. Segmentation for whole lung on each phase was performed in the treatment planning system. Figure 8 shows the fused images on phase 4 (phase 40%Ex) of patient #1 and phase 7 (phase 20%In) of patient #6, respectively. Up to 8 mm spatial difference in diaphragm position [Fig. 8(a)] and tumor contour [Fig. 8(b)] can be observed.

**Figure 9:**
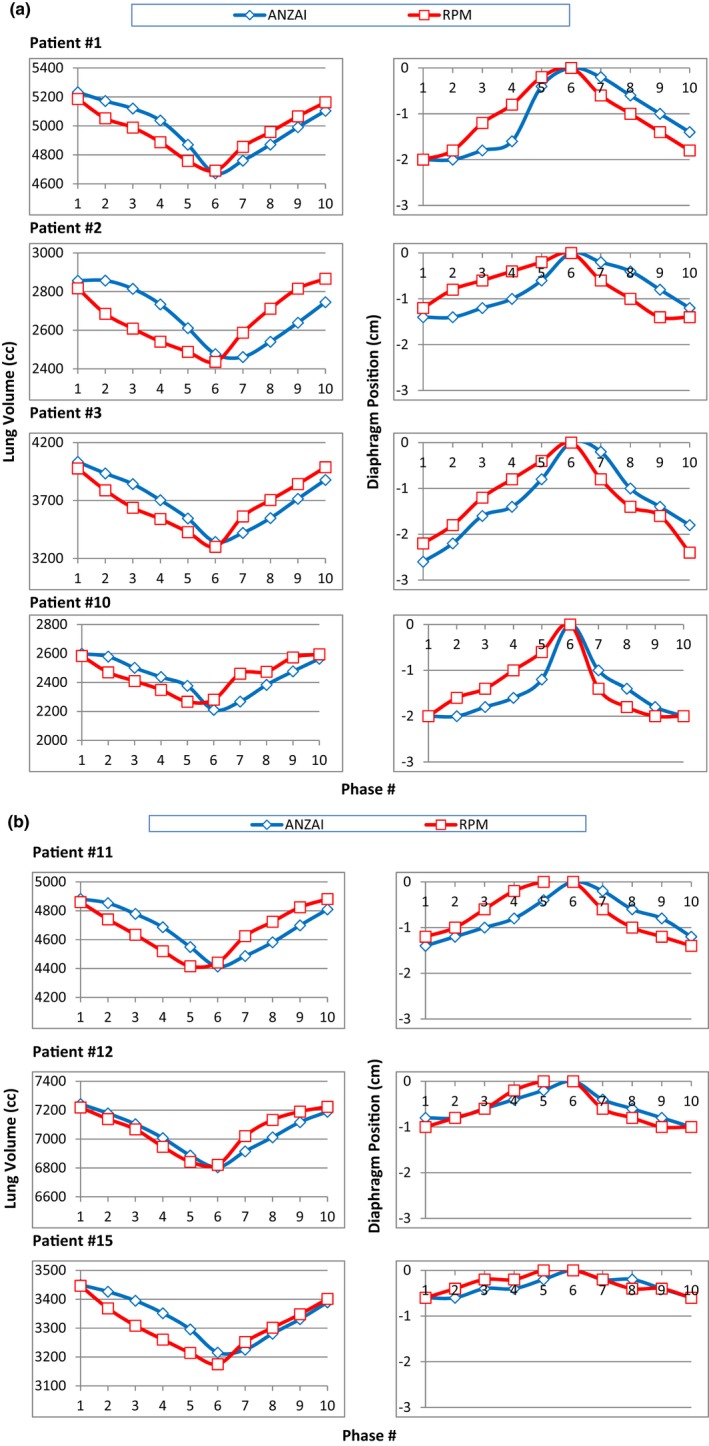
(a) (Left column) the whole‐lung volume vs phase #, and (right column) the diaphragm position (relative to phase #6) vs phase #, measured on 4D images reconstructed using ANZAI (blue diamond) and RPM (red square) data for patient #1, #2, #3 and #10. (b) (Left column) the whole‐lung volume vs phase #, (right column) the diaphragm position (relative to phase #6) vs phase #, measured on 4D images reconstructed using ANZAI (blue diamond) and RPM (red square) data for patient #11, #12 and #15.

The whole‐lung volume and the axial displacement of the diaphragm apex as the function of phase number are shown in Fig. 9. The displacement of the diaphragm apex is measured as the superior–inferior position change relative to the EOE phase (phase 6, or phase 0%In). As the reconstructed axial slices have a thickness of 2 mm, the change in diaphragm position has a step length of 2 mm. The numerical single‐phase differences between ANZAI and RPM measurements in whole‐lung volume and diaphragm position are summarized in Table 2. The single‐phase differences are given as the range of differences over 10 phases. The overall single‐phase differences averaged over the seven patients are −3.7% to +4.1% in whole‐lung volume and −5.1 mm to +4 mm in diaphragm position.

**Table 2:** The range of single‐phase‐differences (ANZAI−RPM) in whole lung volumes and diaphragm positions as shown in Figure 9

Patient #	Single‐phase‐difference in whole lung volume	Single‐phase‐difference in diaphragm apex position (mm)
1	−2% to +3%	−8mm to +4mm
2	−6.3% to +7.9%	−6mm to +6mm
3	−4.2% to +5.6%	−6mm to +6mm
10	−7.8% to +4.9%	−6mm to +4mm
11	−3% to +3.7%	−6mm to +4mm
12	−1.7% to +0.9%	−2mm to +2mm
15	−0.8% to +2.8%	−2mm to +2mm
**Average**	**−3.7% to +4.1%**	**−5.1mm to +4mm**

## 4 DISCUSSIONS

The synchronized respiratory signal acquired by the ANZAI and RPM surrogate systems were found to be in disagreement as characterized by phase shift and shape distortion in respiratory traces. Over the 15 patients, the linear correlation coefficients between ANZAI and RPM data were measured with the lowest values (~0.7) in patients #2, #14 and highest values (>0.9) in patients #5, #6, #7, #12 and #13. Accordingly, the phase shifts are largest in patients #2 (0.3 ± 0.1 s) and #14 (0.27 ± 0.07 s) and smallest in patients #5 (0.15 ± 0.03 s), #6 (0.18 ± 0.03 s), #7(0.13 ± 0.02 s), #12(0.11 ± 0.05 s), and #13(0.11 ± 0.02 s). It may be concluded that phase shift between ANZAI and RPM traces plays a major role in causing the discrepancy that is measured by the linear correlation coefficient.

Possible causes of the phase shift between ANZAI and RPM include the intrinsic asynchony of abdominal movements along the dorsoventral and lateral directions with respiration,^17^ and the underlying latency in signal recording of the two different surrogate systems. It is beyond the scope of this study to investigate the potential causes of the phase shift witnessed. The method using the paired mice to simultaneously initiate recording on both systems is consistent with the realistic scenario in clinical practice in which the start of RPM or ANZAI recording is also controlled by mouse. The impact of the soldering and wiring of the paired mice was investigated before all the measurements on patients and consistent phase shifts between ANZAI and RPM were measured no matter which mouse was clicked to start the simultaneous signal acquisition. Therefore, we can assume the proposed synchronization method has not increased extra uncertainty to the measured phase shift.

The correlation scatter plot (Fig. 4) approximately forms an elliptical distribution. An ellipse fitting is also shown in Fig. 4, using generalized eigenvalue decomposition.^19^ For simplicity, the respiratory trace can be approximated as a periodic function of time. If a 2D state space is augmented with the coordinate consisting of the respiration amplitude at a current time t and a past time t‐Δ (the interval Δ is fixed), the scatter plot in the augmented state space would be distributed on an ellipse.^19^, ^20^ Therefore, such elliptical fitting in the correlation plot also indicates the existence of a phase shift between the ANZAI and RPM data. The fitted ellipse parameters can be used as an alternative way to calculate the mean value of the phase shift.^20^

The trace shape within a full breathing cycle does not match ideally between ANZAI and RPM data. It was found that for all 15 patients, during exhalation the abdominal surface pressure change (ANZAI) has a steeper falloff than the anterior–posterior (AP) displacement (RPM). The behavior during inhalation can be different, with nine patients (patient #1–9) having good match, five patients (patient #10–14) having steeper rise in surface pressure (ANZAI), and one patient (patient #15) having steeper rise in AP displacement (RPM). It implies that the abdominal surface pressure change and surface displacement may have nonlinear response rate to respiratory motion. The mismatch in the trace shape may be attributed to the difference between the two different physical quantities the two surrogates measure.

The phase shift and trace shape discrepancies between ANZAI and RPM together result in different sinogram sorting for 4D image reconstruction. The anatomic changes in images compared phase by phase are measured by the differences in whole‐lung volume and diaphragm position. As shown in Fig. 9, the trends of variation in lung volume and diaphragm position as a function of phase in ANZAI and RPM are similar and can be approximately matched with each other by shifting one phase.

The maximum intensity projection (MIP) and average intensity images were also generated based on the 4D images of ANZAI and RPM, respectively. The differences in MIP and average images were found to be negligible. Figure 9 shows that the ranges of lung volume and diaphragm position over 10 phases are in agreement between ANZAI and RPM. Therefore, the 4D images of ANZAI and RPM consisting of all 10 phases can be equally used for contouring the internal target volume and treatment planning. However, the impact of the discrepancies between ANZAI and RPM occurs when treatment gating with a portion of 4D images used is implemented and the online respiratory monitoring system is different from that used for 4D CT. In such case, the gating window designed in treatment planning based on 4D CT is mismatched with that applied for gated radiation delivery, which may cause errors in dose distribution received by the patient.

## 5 CONCLUSIONS

In this study, the correlation between two respriatory monitoring systems (ANZAI and RPM) was evaluated and discrepancies were found as characterized as phase shift and shape distortion between the respiratory traces. The results indicate that the two external surrogates have nonequivalent correlation to internal organ motion with respiration. Mixed use of the two surrogates for 4D CT and gated treatment should be avoided as the same gating window does not match on different surrogates and potential errors in dose distribution received by the patient may be thus caused.

## CONFLICT OF INTEREST

The authors have no conflicts of interest to disclose.
